# Multifocal Electroretinography Changes in Patients with Late-Stage Age-Related Macular Degeneration (AMD) After Smaller-Incision New-Generation Implantable Miniature Telescope (SING IMT): A Case Series

**DOI:** 10.3390/jpm14121119

**Published:** 2024-11-25

**Authors:** Luca Landini, Giacomo Boscia, Faustino Vidal-Aroca, Alfredo Niro, Valentina Pastore, Marina Piepoli, Pasquale Viggiano, Maria Oliva Grassi, Ermete Giancipoli, Maria Grazia Pignataro, Giovanni Alessio, Marc H. Levy, Giancarlo Sborgia, Francesco Boscia

**Affiliations:** 1 Department of Translational Biomedicine Neuroscience, University of Bari “Aldo Moro”, 70124 Bari, Italy; g.boscia@studenti.uniba.it (G.B.); valentinapastore@hotmail.it (V.P.); marina.piepoli@uniba.it (M.P.); pasquale.viggiano@uniba.it (P.V.); m.pignataro12@studenti.uniba.it (M.G.P.); giovanni.alessio@uniba.it (G.A.); giancarlo.sborgia@policlinico.ba.it (G.S.); francesco.boscia@uniba.it (F.B.); 2 Department of Medical Affairs, Samsara Vision Ltd., Via Umbria 24, 20068 Milan, Italy; vidal.aroca@gmail.com; 3 Eye Clinic, “SS. Annunziata” Hospital, ASL TA, 74121 Taranto, Italy; alfred.nir@tiscali.it; 4 Department of Ophthalmology, Policlinico Riuniti Foggia, University of Foggia, 71100 Foggia, Italy; ermete.giancipoli@unifg.it; 5 Eye Clinic, Sarasota Retina Institute, Sarasota, FL 34239, USA; marchlevy@aol.com

**Keywords:** late-stage age-related macular degeneration (AMD), geographic atrophy, visual impairment, implantable ophthalmic device, intraocular telescope (IOT), low vision, smaller-incision new-generation implantable miniature telescope, SING IMT, multifocal electroretinography (mfERG)

## Abstract

The smaller-incision new-generation implantable miniature telescope (SING IMT) represents an advancement over the previous model, WA-IMT, serving as a unilateral prosthetic device for patients with late-stage age-related macular degeneration (AMD). **Purpose:** This study aims to report changes in multifocal electroretinography (mfERG) 6 months post–SING IMT implantation. **Methods:** In this case series, we prospectively evaluated a cohort of phakic patients with late-stage AMD who underwent SING IMT implantation at the Ophthalmology Unit, University of Bari Aldo Moro, Italy. We assessed best-corrected distance visual acuity (BCDVA) and best-corrected near visual acuity (BCNVA) preoperatively and at 6 months postoperatively. Additionally, mfERGs were conducted using Retimax (CSO, Florence, Italy). **Results:** All four treated patients showed an increase in both BCDVA and BCNVA at the 6-month follow-up. Additionally, all eyes demonstrated increased P1 density at this time point, with the greatest augmentation observed at the central fixation point, gradually diminishing across the five concentric rings. While all patients displayed a general increase in P1 amplitude, the third patient exhibited a slight decrease in the foveal region. **Conclusions:** In this case series with four cases, the new generation implantable miniature telescope, SING IMT, demonstrates promising results in enhancing mfERG parameters in patients with late-stage AMD. Six months post-surgery, we observed an augmentation in both P1 density and amplitude, predominantly at the fixation point and gradually tapering in the surrounding concentric rings.

## 1. Introduction

Age-related macular degeneration (AMD) is one of the leading causes of vision impairment in aging populations within developed countries. In advanced stages, AMD can take a “wet” form, characterized by macular neovascularization (MNV) and subsequent macular scarring, and a “dry” form characterized by geographic atrophy (GA) [1]. At present, the prevalence of late-stage AMD is estimated to be between 1.1% and 3.9% [2]. Due to its involvement of the macula, AMD enforces significant disability, with the United States alone estimating the cost of visual impairment stemming from AMD to be USD 98 billion [1,3]. Consequently, novel treatment modalities, both surgical and nonsurgical, are actively under development to alleviate this burden. In efforts to enhance the quality of life (QoL) for individuals afflicted with late-stage AMD, various magnification devices, including low-vision aids, have been devised. However, external aids have limitations, owing to their bulkiness and the ensuing reduction in visual field, often accompanied by vestibular complications [4].

The Galilean implantable miniature telescope (IMT, Vision Care Ophthalmic Technologies, Saratoga, CA, USA) marked the initial foray into implantable intraocular telescopes (IOTs) tailored for patients experiencing visual impairment due to bilateral, late-stage AMD. However, these devices were encumbered by a reduction in endothelial cell density (ECD) attributed to surgical maneuvers [5,6,7].

This issue has been mitigated with the advent of the smaller-incision new-generation implantable miniature telescope (SING IMT), representing the second iteration of the IMT. The SING IMT retains the optics of its precursor but boasts a smaller diameter and flexible silicone haptics, enabling a reduced incision size (8 mm compared to 13 mm) and decreased ECD loss. Both the IMT and SING IMT function via a compound lens system comprising an anterior positive lens and a posterior negative lens. This system, in tandem with the cornea, operates as a telephoto device with a focusing distance of 0.5 m and a 2.7× angular magnification, delivering a high-resolution magnified image onto 54 degrees of the retina with a 20-degree nominal field of view [7,8].

Multifocal electroretinography (mfERG) serves to record the electrophysiological responses coming from various retinal regions in response to a light stimulus, constituting a valuable tool for assessing retinal function. The waveforms of mfERG exhibit a biphasic nature, comprising a negative initial component (N1) corresponding to responses from the “off” bipolar cells, and a subsequent positive peak (P1) indicative of activation of the “on” bipolar cells. Several factors, including age, lens density, and pupil size, influence the quality of the mfERG recordings [9,10]. This case series aims to elucidate the changes in mfERG following SING IMT implantation.

## 2. Materials and Methods

This case series retrospectively evaluated a cohort of phakic patients with late-stage AMD who underwent SING IMT implantation at the Ophthalmologic Unit, University of Bari Aldo Moro, Italy. Consistent with our institution’s guidelines and in accordance with Good Clinical Practice (GCP) and the principles of the Declaration of Helsinki, we notified the Ethics Committee of this study, as we have done for other retrospective studies. This process ensures that our work aligns with institutional standards and international guidelines for ethical research practices, even in the absence of formal approval requirements. Indeed, considering Italian law, there is no need to request an opinion from any Ethics Committee. All patients provided written informed consent for both the surgical procedure and the processing of personal data.

### 2.1. Patient Selection

The inclusion and exclusion criteria for this study aligned with surgical indications for SING IMT implantation. Patients were required to be aged 55 years or older with bilateral late-stage AMD (GA or disciform scar) causing central visual acuity loss. Criteria for the implant eye included being phakic with a cataract and having an endothelial cell density (ECD) greater than 1600 cells/mm^2^, anterior chamber depth (ACD) of at least 2.5 mm, intraocular pressure (IOP) below 22 mmHg, and bilateral best-corrected distance visual acuity (BCDVA) ranging from 0.6 to 1.6 LogMAR. The implant eye needed a potential improvement of at least five ETDRS chart letters, verified via an External Telescope Simulator (ETS 3.0X-FF; VisionCare Ophthalmic Technologies Ltd., Petah Tikva, Israel). If both eyes met criteria, the eye with greater letter gain using ETS was selected. Additionally, patients needed to be capable, willing, and motivated for postoperative vision training and rehabilitation.

### 2.2. Patients’ Evaluation

Patients underwent evaluation both preoperatively and 6 months post-surgery. To assess whether they met the indication criteria, BCDVA was tested using ETDRS charts at a distance of 4 m, and the TS was utilized to determine potential visual gain (at least 5 letters) post-implantation. Best-corrected near visual acuity was also assessed using critical print size (CPS) with MNREAD Acuity Charts (Optelec, US Inc., Clearwater, FL, USA) at a 40 cm distance. By ophthalmoscopy, the anterior segment and lens status were assessed. Fundus examination and SD-OCT scans (Spectralis; Heidelberg Engineering; Heidelberg, Germany) were conducted to confirm the presence of GA or a disciform scar and to rule out active neovascularization or other retinal comorbidities. Endothelial cell density (ECD) was measured using endothelial microscopy (Perseus, CSO, Florence, Italy), axial length and ACD were determined using ocular biometry (IOL Master 500, Carl Zeiss Meditec AG, Jena, Germany), and intraocular pressure (IOP) was measured using Perkins applanation tonometry.

### 2.3. Multifocal Electroretinography (mfERG) Assessment

mfERGs were conducted both preoperatively and 6 months post-surgery using Retimax (CSO, Florence, Italy) in accordance with ISCEV standards [11]. With the pupil fully dilated, a positive loop electrode was positioned on the previously anesthetized conjunctiva of the examined eye, a negative electrode on the lateral canthus of the same eye, and a common electrode was affixed to the forehead. The fellow eye was occluded, and the patient was positioned 30 cm from the screen displaying a board of 61 hexagons, with instructions to maintain fixation on a central target throughout the duration of the test. Evaluation was conducted across five concentric rings, with the average response within each ring calculated as the sum of responses from each hexagon divided by the number of hexagons within the ring. Parameters assessed included P1 amplitude, defined as the difference between the most negative voltage of the N1 wave and the highest voltage of the P1 wave, and P1 density, representing the normalization of response per unit area of retina, considering variations in hexagon sizes from the center to the periphery.

### 2.4. Surgical Technique

All four surgeries were performed by an experienced vitreoretinal surgeon (F.B.) under local anesthesia with a retrobulbar block administered 10 min prior to surgery. The initial step involved creating a 6.5 mm circular capsulorhexis and performing a nuclear cross cut using a Femtolaser (FEMTO LDV Z8, Ziemer Group, Port, Switzerland). Conjunctival peritomy was carried out in the upper quadrant, and scleral bleeding was controlled with bipolar diathermy forceps. A 2.4 mm multiplanar limbal tunnel was then created at the 12 o’clock position, followed by filling the anterior chamber with a cohesive ophthalmic viscosurgical device (OVD) and removal of the precut capsulorhexis with capsulorhexis forceps. Subsequent steps included hydrodissection, nucleus rotation, phacoemulsification, and cortical removal. The anterior chamber and capsular bag were once again filled with cohesive OVD, and the limbal tunnel was enlarged to 8 mm. The preloaded Tsert delivery system was inserted with the tip of the injector positioned at the level of the capsulorhexis and angulated at approximately 45° on the iris plane. After device insertion, the position of each haptic was verified, the limbal wound was sutured with separate 10/0 nylon monofilament sutures, and the OVD was removed. A basal superior iridectomy was performed, followed by suturing of the conjunctiva to conclude the procedure.

## 3. Results

### 3.1. Patient 1

**Figure 1:**
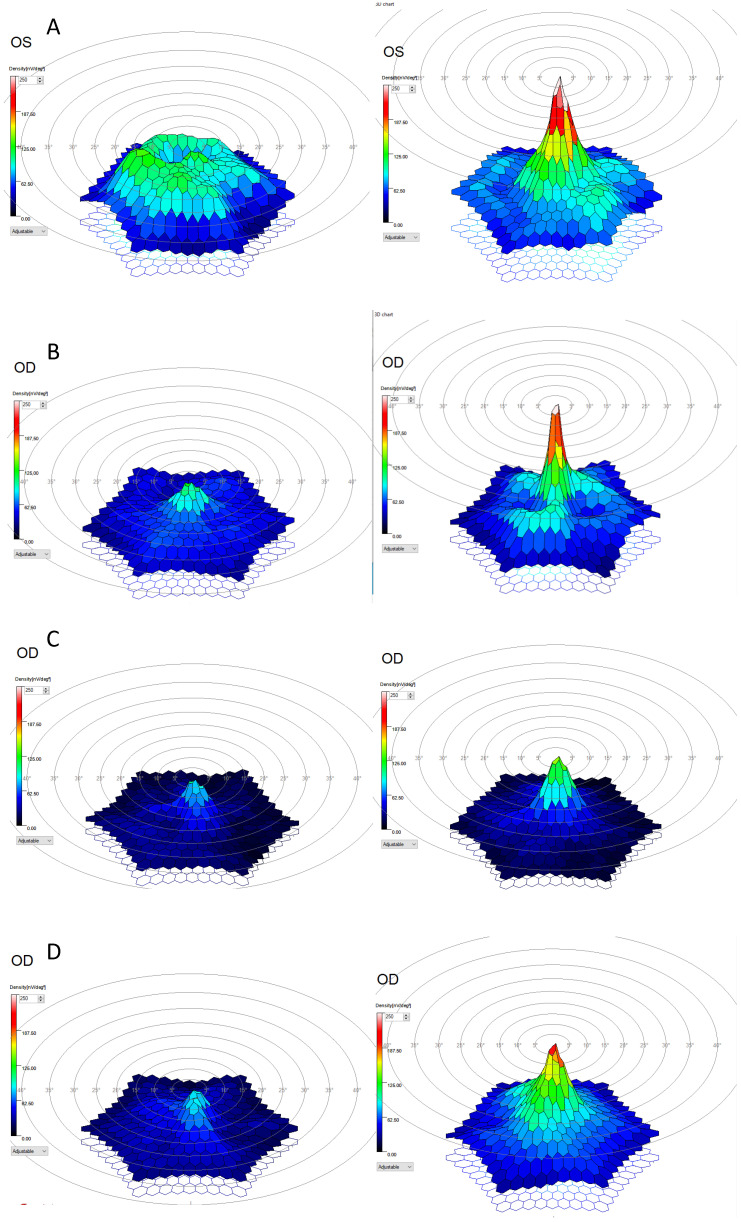
Changes in P1 density 3D map. On the left before surgery, on the right 6 months after SING IMT implantation. (**A**) Patient 1. (**B**) Patient 2. (**C**) Patient 3. (**D**) Patient 4.

An 86-year-old woman underwent implantation in the left eye. Preoperatively, the best-corrected distance visual acuity (BCDVA) was 6 letters (0.9 LogMAR), and the best-corrected near visual acuity (BCNVA) was 29 characters per second (cps). At the 6-month follow-up after SING IMT implantation, the BCDVA improved to 30 letters (0.6 LogMAR), and the BCNVA improved to 9.3 cps. Intraocular pressure (IOP) was recorded at 12 mmHg (Table 1). In the foveal region, the multifocal electroretinogram (mfERG) P1 density increased from 91.87 nV/deg^2^ to 265 nV/deg^2^, and the amplitude of the P1 wave increased from 0.51 µV to 0.99 µV (Table 2 and Figure 1A).

### 3.2. Patient 2

An 82-year-old woman underwent implantation in the right eye. Preoperatively, BCDVA was 2 letters (1.36 LogMAR), and BCNVA was 74 cps. Six months post-implantation, BCDVA improved to 25 letters (0.6 LogMAR), and BCNVA improved to 22 cps (Table 1). The IOP was recorded at 11 mmHg. In the foveal region, the mfERG P1 density increased from 123.34 nV/deg^2^ to 307.37 nV/deg^2^, and the amplitude of the P1 wave increased from 0.62 µV to 1.48 µV (Table 2 and Figure 1B).

### 3.3. Patient 3

An 84-year-old male underwent implantation in the right eye. Preoperatively, BCDVA was 2 letters (1.36 LogMAR), and BCNVA was 74 cps. At the 6-month follow-up, BCDVA improved to 15 letters (0.8 LogMAR), and BCNVA improved to 22 cps. The IOP was recorded at 11 mmHg. In the foveal region, the mfERG P1 density increased from 79.19 nV/deg^2^ to 88.1 nV/deg^2^, while the amplitude of the P1 wave slightly decreased from 0.66 µV to 0.64 µV (Table 2 and Figure 1C).

### 3.4. Patient 4

An 82-year-old woman underwent implantation in the right eye. Preoperatively, BCDVA was 5 letters (1 LogMAR), and BCNVA was 23 cps. At the 6-month follow-up, BCDVA improved to 29 letters (0.5 LogMAR), and BCNVA improved to 15 cps. The IOP was recorded at 12 mmHg. In the foveal region, the mfERG P1 density increased from 34.71 nV/deg^2^ to 220.76 nV/deg^2^, and the amplitude of the P1 wave increased from 0.17 µV to 1.18 µV (Table 2 and Figure 1D).

No intraoperative, aborted surgeries or postoperative complications were observed in any patient, and none required IOP-lowering medications post-surgery.

## 4. Discussion

To the best of our knowledge, this is the first description of mfERG changes following SING IMT implantation. We observed an increase in P1 density at 6 months in all four treated patients. This increase was most pronounced at the center of fixation, decreasing rapidly in each of the five concentric rings (Table 2 and Figure 1). Additionally, all patients exhibited a general increase in P1 amplitude, although the third patient showed a slight decrease in the foveal region (Table 2).

Multifocal electroretinography (mfERG) has proven effective in detecting retinal dysfunction in AMD patients and signaling progression to advanced disease stages [12]. Indeed, late-stage AMD has been associated with lower response rates compared to the healthy population [13].

Previous studies have reported the efficacy of SING IMT in improving both BCDVA and BCNVA. Toro, Savastano, and Mastropasqua et al. demonstrated an increase in both distance and near visual acuity 3 months after SING IMT implantation, along with reduced endothelial cell density loss compared to the first-generation IMT [8,14,15].

Subsequently, Sasso et al. reported increased BCDVA following SING IMT implantation, as well as improved reading acuity and speed 24 weeks post-surgery [16].

Consistent with these findings, our results showed improvements in both BCDVA and BCNVA at 6 months post-surgery (Table 1). Furthermore, mfERG assessment revealed improvements in both P1 density and P1 amplitude (Figure 1 and Table 2).

The SING IMT provides 2.7× angular magnification, resulting in a high-resolution enlarged image over 54 degrees of the retina with a 20-degree field of view [7,17]. No alteration in light vergence or incidence on photoreceptors occurs compared to the crystalline lens, suggesting no influence on the Stiles–Crawford effect. However, it is conceivable that the enlarged image, stimulating a greater retinal area for the same central visual field, may enhance retinal cell responses to mfERG registration. This enhancement in retinal function would be in accordance with the findings of the IMT002 Study that demonstrated that fellow eyes that underwent routine cataract surgery only gained 2 letters on the ETDRS chart while the telescope ones (IMT first generation) gained 3.2 lines. Although confirming this speculation exceeds the scope of our study and would require further investigation, we also hypothesize that cataract extraction may contribute to the improvement in mfERG parameters. Literature indicates that nuclear cataracts may affect mfERG topography, although existing studies only include patients without ocular diseases other than cataracts [9,18]. Thus, after cataract extraction, the increased and less scattered light entering the eyes may find a healthy retina and photoreceptors to receive it. In our case, patients had late-stage AMD affecting central photoreceptors, hence a minor increase in central mfERG density and amplitude was expected. Yavas et al. demonstrated reduced responses in late-stage AMD patients compared to a healthy group [13]. Unfortunately, no publications compare mfERG before and after cataract surgery in AMD eyes, so further studies confronting mfERG pre- and postop IMT and routine cataract surgery in the contralateral eye or in a control group will be necessary to support these findings.

The main limitations of our study include a small patient sample and the absence of a control group. Additionally, this is a single-center study enrolling only Caucasian patients. Another important limitation, as previously noted, is the inclusion of patients with dense nuclear cataracts. These cases present a potential bias, as dense cataract extraction may play a significant role in enhancing mfERG response, rather than solely the SING IMT implantation. This consideration is crucial when interpreting mfERG results post–SING IMT implantation. Further studies are needed to elucidate the role of SING IMT on mfERG and changes in mfERG following cataract extraction in patients with late-stage AMD. Nevertheless, the observed improvements in visual acuity and mfERG parameters suggest that SING IMT may offer significant benefits, potentially enhancing visual function and quality of life for patients with late-stage AMD. Although our results are limited by a small sample size and short follow-up period, mfERG may be a valuable tool for monitoring retinal status after surgery and throughout follow-up in patients undergoing SING IMT implantation.

## 5. Conclusions

In conclusion, in our sample of four cases, there is a significant increase in P1 density and amplitude at the 6-month follow-up, with the greatest enhancement observed at the central fixation point. These results are consistent with previous reports on the efficacy of SING IMT in improving both best-corrected distance visual acuity (BCDVA) and best-corrected near visual acuity (BCNVA). The SING IMT, with its 2.7× angular magnification, appears to enhance retinal cell responses, potentially due to the increased retinal area stimulated by the enlarged image. This enhancement in mfERG parameters may translate to several potential patient benefits, including improved visual acuity, better overall visual performance, and an enhanced quality of life through an improved ability to perform daily activities. However, further studies with larger samples and control groups are necessary to confirm these findings and to explore the potential impact of cataract extraction on mfERG parameters in patients with late-stage AMD.

## Figures and Tables

**Table 1:** Improvements in BCDVA and BCNVA at 6 months post–SING IMT implantation for all four patients.

Patient	BCDVA (Letters)		BCNVA (CPS)		IOP (mmHg)	
	Preoperative	Postoperative	Preoperative	Postoperative	Preoperative	Postoperative
1	6	30	29	9,3	18	12
2	2	25	74	22	16	11
3	2	15	74	22	18	11
4	5	29	23	15	15	12

**Table 2:** Increase in P1 density at 6 months post–SING IMT implantation across five concentric rings. With 1 corresponding to the center of fixation and 5 the most peripheral one. The value reported is the mean of the values of every hexagon in that ring.

Patient	Concentric Rings																			
	Density Preoperative					Density Postoperative					P1 Preoperative					P1 Postoperative				
	1	2	3	4	5	1	2	3	4	5	1	2	3	4	5	1	2	3	4	5
1	91.87	10.71	4.96	4.55	0.69	265	64.7	17.7	6.94	6.26	0.51	0.08	0.18	0.17	0.14	0.99	0.47	−0.01	0.12	0.2
2	123.34	27.06	19.65	11.68	3.53	307	26.4	14.1	8.7	8.23	0.62	0.16	0.2	0.15	0.08	1.48	0.24	0.38	0.12	0.09
3	79.19	28.25	5.13	1.57	12.42	88.1	55.5	−1.5	4.81	1.3	0.66	0.05	0.08	−0.01	0.31	0.64	0.41	0.11	0.17	−0.12
4	34.71	37.60	28.15	17.21	15.27	220.76	22.01	15.06	12.61	0.00	0.17	0.22	0.30	0.27	0.33	1.18	0.33	0.19	0.22	0.23

## Data Availability

The datasets generated during and/or analysed during the current study are available from the corresponding author on reasonable request.
